# Hepcidin is described as the master regulator of iron: could its removal by CRRT lead to iron dysmetabolism in the critically ill?

**DOI:** 10.1186/s13054-020-03295-6

**Published:** 2020-09-22

**Authors:** Patrick M. Honore, Leonel Barreto Gutierrez, Luc Kugener, Sebastien Redant, Rachid Attou, Andrea Gallerani, David De Bels

**Affiliations:** ICU Department, Centre Hospitalier Universitaire Brugmann, Brugmann University Hospital, Place Van Gehuchtenplein, 4, 1020 Brussels, Belgium

Litton et al. noted that many of the risk factors for iron deficiency are also risk factors for developing a critical illness, and consequently, iron deficiency is likely to be over-represented in critically ill patients [1]. Hepcidin is described as the master regulator of iron that determines the severity and duration of an iron-restricted state [1]. Insufficient hepcidin levels are central to iron overload while hepcidin excess leads to iron restriction [1]. A persistent state of iron dysmetabolism not only predisposes a vulnerable population to decreased erythropoiesis, but also has implications for the risk of nosocomial infection and critical illness-associated cognitive, neuromuscular, and cardiopulmonary dysfunction [1]. A recent cohort study of 807 critically ill patients with acute kidney injury (AKI) requiring renal replacement therapy (RRT) found that both higher plasma concentrations of catalytic iron and lower concentrations of hepcidin are associated with increased mortality [2]. A key question arising from this study is to what extent RRT contributes to iron dysmetabolism in critically ill patients. Fifty percent of critically ill patients develop AKI and 25% require RRT [3]. Hepcidin and pro-hepcidin have molecular weights of 2700 Da and 10,000 Da, respectively, and, therefore, may be removed by continuous RRT (CRRT), which uses membranes with a cut-off of 35,000 Da [4]. The protein-bound fraction of hepcidin is about 40% [4] which does not impede its elimination by convection or diffusion [5]. It has been demonstrated that maintenance dialysis with both super-flux polysulphone (PS) and acrylonitrile 69 (AN69) membranes similarly removed hepcidin [5]. Hemodialysis with PS membranes may achieve a high removal ratio of hepcidin by enhanced diffusion performance and an increased clearance of small molecule solutes, while AN69 membranes may remove hepcidin by adsorption [5]. Indeed, hepcidin can be removed by diffusion, while in the case of pro-hepcidin, convection is the main mechanism [5]. There are other factors to consider beyond RRT, and we should remain prudent regarding the therapeutic implications of RRT modalities at this early stage. The timing of iron administration in patients admitted to intensive care may also be a strong determinant of whether the benefits outweigh the risks [1]. In addition, other factors such as bleeding in anticoagulated patients or clotting of dialysis membranes may limit iron accumulation in critically ill patients. A better understanding of the epidemiology and outcomes of iron metabolism during critical illness is needed before designing interventional studies looking at iron metabolism during CRRT.

## Data Availability

Not applicable.

## Abbreviations

RRT: Renal replacement therapy
AKI: Acute kidney injury
Da: Daltons
CRRT: Continuous renal replacement therapies
PS: Polysulphone
AN69: Acrylonitrile 69
